# Exploring the interactions of short RNAs with the human 40S ribosomal subunit near the mRNA entry site by EPR spectroscopy

**DOI:** 10.1093/nar/gkz1039

**Published:** 2019-11-14

**Authors:** Alexey A Malygin, Olesya A Krumkacheva, Dmitri M Graifer, Ivan O Timofeev, Anastasia S Ochkasova, Maria I Meschaninova, Alya G Venyaminova, Matvey V Fedin, Michael Bowman, Galina G Karpova, Elena G Bagryanskaya

**Affiliations:** 1 Institute of Chemical Biology and Fundamental Medicine SB RAS, Pr. Lavrentjeva 8, Novosibirsk 630090, Russia; 2 N. N. Vorozhtsov Novosibirsk Institute of Organic Chemistry SB RAS, Pr. Lavrentjeva 9, Novosibirsk 630090, Russia; 3 Novosibirsk State University, Pirogova Str. 2, Novosibirsk 630090, Russia; 4 International Tomography Center SB RAS, Institutskaya Str. 3a, Novosibirsk 630090, Russia; 5 University of Alabama, Tuscaloosa, AL 35487-0336, USA

## Abstract

The features of previously unexplored labile complexes of human 40S ribosomal subunits with RNAs, whose formation is manifested in the cross-linking of aldehyde derivatives of RNAs to the ribosomal protein uS3 through its peptide 55–64 located outside the mRNA channel, were studied by EPR spectroscopy methods. Analysis of subatomic 40S subunit models showed that a likely site for labile RNA binding is a cluster of positively charged amino acid residues between the mRNA entry site and uS3 peptide 55–64. This is consistent with our finding that the 3′-terminal mRNA fragment hanging outside the 40S subunit prevents the cross-linking of an RNA derivative to this peptide. To detect labile complexes of 40S subunits with RNA by DEER/PELDOR spectroscopy, an undecaribonucleotide derivative with nitroxide spin labels at terminal nucleotides was utilized. We demonstrated that the 40S subunit channel occupancy with mRNA does not affect the RNA derivative binding and that uS3 peptide 55–64 is not involved in binding interactions. Replacing the RNA derivative with a DNA one revealed the importance of ribose 2′-OH groups for the complex formation. Using the single-label RNA derivatives, the distance between the mRNA entry site and the loosely bound RNA site on the 40S subunit was estimated.

## INTRODUCTION

**Figure 1.**
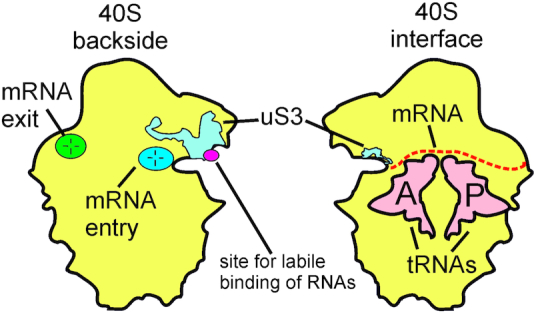
Schematic representation of the 40S subunit of the eukaryotic ribosome and its functional sites. The mRNA binding channel is located on the interface side of the 40S subunit (which in the 80S ribosome faces the 60S subunit). In this channel, mRNA codons interact with the anticodons of tRNA molecules bound at two ribosomal sites, the P (peptidyl) site for tRNA with a nascent peptide chain, and the A (aminoacyl) site for newly coming aminoacyl-tRNA carrying an aminoacyl residue to be added to this chain. The mRNA entry and exit sites located on the backside of the 40S subunit, and the site for the labile binding of unstructured RNAs with the participation of the ribosomal protein uS3, identified in our previous study, are also marked (6).

Ribosomes are giant cellular machines that translate genetic information encoded in nucleotide sequences of messenger RNAs (mRNAs) into amino acid sequences of proteins. Ribosomes consist of two subunits denoted by their sedimentation coefficients: in eukaryotes, small (40S) and large (60S) subunits, while the full ribosome is known as the 80S one. The 40S subunit contains a binding channel for mRNAs bearing genetic information transcribed from the genome. The channel includes the decoding site where trinucleotide segments (codons) of the mRNA are recognized by complementary anticodons of transfer RNAs (tRNAs) bearing amino acid residues for protein synthesis (Figure 1). The large 60S subunit contains the catalytic center where the amino acid residues form peptide bonds to elongate the growing protein chain. Both subunits have binding sites for tRNA molecules, translation factors and specific accessory proteins necessary for the synthesis of polypeptides.

Deciphering the structure of the eukaryotic ribosome by various biochemical and structural approaches, particularly X-ray crystallography and high-resolution cryo-electron microscopy (cryo-EM), has identified many of the molecular contacts between the ribosome and the traditional participants of protein synthesis (for review, see 1 and 2). In addition, photoactivatable RNA analogues able to cross-link to proteins have revealed interactions, never detected in cryo-EM studies, between 80S ribosomes or 40S subunits and unstructured RNAs outside the mRNA binding channel (3–7). Rather efficient cross-linking to the small subunit ribosomal protein uS3 occurs in binary mixtures with 80S ribosomes or 40S subunits, when the RNA derivatives are not fixed in the mRNA binding channel by their interaction with a cognate tRNA. Cross-linking to uS3 is observed with various photoactivatable aryl azide derivatives of RNA and DNA oligomers (3–5), as well as 3′-terminal dialdehyde derivatives of RNA oligomers (6,7). The region of uS3 involved in the cross-link has been mapped to peptide 55–64 (most likely, to its K62, which is the only lysine in this peptide able to react with aldehydes). This uS3 peptide 55–64 region lies outside the mRNA binding channel on the exposed 40S subunit surface near the mRNA entry site (6) (Figure 1). Despite efficient cross-linking of oligonucleotide derivatives to uS3, their binary complexes with human 80S ribosomes or 40S subunits are very labile and barely detectable by the nitrocellulose filtration technique, which is usually used to examine the binding of labeled ligands to ribosomes (3–6). The rather high yield of cross-links generated from such labile complexes most likely results from the removal of the cross-linked products from the binding equilibrium and the rapid re-establishment of the equilibrium between the remaining ribosomes and the unreacted derivatives. The uS3 peptide 55–64 is available for binding to short RNAs at almost all stages of translation, except for the early one, when the 48S pre-initiation complex is formed, which precedes loading of the correct mRNA into the 40S subunit mRNA binding channel (7). Based on these data, the above peptide might be involved in the regulation of translation (7). However, the exact role played by the ability of 40S ribosomal subunits to interact with short unstructured nucleic acids via the exposed uS3 peptide remains unclear, and almost nothing is known about these complexes.

Electron paramagnetic resonance (EPR) spectroscopy using RNA derivatives bearing spin labels at selected locations allows the study of RNA binding to ribosomes (8,9). In our previous work, we have shown that the use of an RNA derivative simultaneously carrying two nitroxide spin labels at the 3′- and 5′-termini enables direct measurements of labile binary complexes of oligoribonucleotides with human ribosomes whose formation is provided by 40S subunits (9). Complexes of this type could not be studied using cryo-EM or X-ray crystallography, the most powerful approaches traditionally used to investigate various ribosomal complexes (e.g. see 10 and 11), since they are applicable to stable ones where ligands are tightly fixed on the ribosomes at the respective binding sites. EPR spectroscopy utilizing spin-labeled RNAs allows distinguishing between free RNAs and ribosome-bound ones in the same solution, as well as between RNAs bound at different ribosomal sites. This owes to the distinctive EPR parameters (e.g. <r_DEER_>) inherent in spin-labeled RNAs in these states, which makes it a good tool for examining labile interactions of RNAs with ribosomes.

Here, we applied DEER/PELDOR spectroscopy to gain information on the labile complexes of the 40S subunit with unstructured RNAs. The doubly spin-labeled derivative of the undecaribonucleotide UCUUGAUUAGA was used to monitor the intramolecular spin-spin distances in the free oligomer and the ribosome-bound one. A similar derivative of its deoxy-analogue TCTTGATTAGA helped to determine the role of ribose 2′-OH groups in the interaction of single-stranded nucleic acids with 40S subunits. The utilization of RNA derivatives containing a single spin label at the 3′-terminal nucleotide made it possible to determine the intermolecular spin-spin distance between two oligomers simultaneously bound to the 40S ribosomal subunit. One was fixed as an mRNA analogue in the 40S subunit channel, and another was located at the site of loosely binding of RNAs. Based on the results obtained using DEER/PELDOR spectroscopy in combination with traditional biochemical approaches, and on the available information on the human 80S ribosome structure, we determined the 40S subunit area where the site for labile binding of RNAs could be located and suggested the functional role of this binding.

## MATERIALS AND METHODS

### Spin labeled derivatives of nucleic acids

The undecaribonucleotide UCUUGAUUAGA with ethylene diamine attached to the C5 and C8 atoms of the 5′-terminal uridine and 3′-terminal adenosine residues, respectively, was synthesized and purified as described (12). The 3D structure of this oligoribonucleotide was predicted using RNAComposer (13) on the online RNA structure modeling server (http://rnacomposer.cs.put.poznan.pl) in interactive mode. The oligoribonucleotides GACUUCAACAAACACAACU, AAUAAAUAU and AUCU, with the C5 atom of their 3′-terminal uridines bearing an ethylene diamine residue, were prepared in a similar way. Unmodified oligoribonucleotides were prepared as described (7).

The 3-carboxy-2,2,5,5-tetramethyl-2,5-dihydro-1H-pyrrol-1-oxyl succinimidyl ester (NHS-M2) spin label was prepared as described in (14) without deuteration (8,9). A doubly spin-labeled RNA derivative (MR11) labeled at the 5′- and 3′-terminal bases was obtained from the above undecaribonucleotide by treatment with NHS-M2 and subsequent purification (9). The doubly spin-labeled undecadeoxyribonucleotide TCTTGATTAGA with spin labels on the 3′- and 5′-terminal phosphates (MD11, a DNA analogue of MR11) was synthesized as described earlier (15).

Derivatives of oligoribonucleotides GACUUCAACAAACACAACU, AAUAAAUAU and AUCU spin labeled at their 3′-terminal U were prepared as described (9). A derivative of pAAUAAAUAU with a 5′-terminal spin label was prepared according to (8). The spin labels content in oligonucleotides was examined by EPR (Supplementary Figures S2, S3 and Supplementary Tables S1 and S2) and by UV–vis spectroscopy. Good agreement between concentration of oligonucleotides and corresponding concentration of labels was obtained indicating that spin-labeling procedures were performed successfully.

### 40S ribosomal subunits: free or cross-linked to an oligoribonucleotide derivative

40S ribosomal subunits were isolated from human placenta that that was never subjected to a freezing as described (16). The subunits were resuspended in D_2_O at a concentration of 35–45 pmol/μl in the final step and stored in liquid nitrogen in small aliquots, so that each was subjected to only one thawing. The subunits were reactivated prior to use by incubation in binding buffer A (50 mM Tris–HCl, pH 7.5, 100 mM KCl, 13 mM MgCl_2_ and 0.5 mM EDTA in D_2_O) at 37°C for 10 min. Where indicated, the reactivated subunits were treated with nonaribonucleotide AAUAAAUAU having a 3′-terminal ribose oxidized to dialdehyde for cross-linking to uS3 (6,7). The initial reaction mixture contained 0.4 nmol of 40S subunits and 7.5 nmol of the derivative in 46 μl of buffer A. After the treatment, the subunits were pelleted by centrifugation at 48,000 rpm for 17 h in a Beckman SW60 rotor. The pelleted 40S subunits were resuspended in D_2_O at a concentration of ∼30 μM. The resulting 40S subunits were used in binding experiments with MR11. The presence of the cross-linked uS3 in this 40S sample was confirmed by western blot analysis of the total ribosomal protein isolated from it using specific antibodies Anti-RPS3 ab140676 against rabbit uS3 purchased from Abcam (7). The amount of cross-linked uS3 was calculated from scans of the western blot using the ChemiDoc XRS imaging system (Bio-Rad) and assuming that the intensities of the bands of the cross-linked uS3 and of the unmodified uS3 total 100%. Statistical processing of the results was carried out using the GraphPad Prism program.

### Complexes of 40S subunits with pre-bound mRNA and tRNA and their cross-linking to an oligoribonucleotide derivative

The scheme of formation of ribosomal complexes containing an mRNA analogue and tRNA at the P site was based on two well-known ideas. First, in the absence of translation factors, tRNAs have a higher affinity for the ribosomal P site than to other sites, and, secondly, the tight binding of a short mRNA analogue to the ribosome occurs only in the presence of the tRNA cognate to one of its triplets (e.g. see 3–7). Accordingly, ternary complexes of 40S subunits (9 pmol) with tRNA^Phe^ (45 pmol) and short mRNAs UUCGACAACAAACACAACAAACA or UUCGACAACAAACACAACAAACGAAUAACA (9 pmol) were obtained by incubating the reactivated subunits with the corresponding components in buffer A. First, subunits were incubated with tRNA^Phe^ at 37°C for 20 min, then mRNA was added and the incubation was continued at 23°C for 1 h. The final volume of the mixture was 16 μl. After the ternary complex with mRNA and tRNA was formed, 9 pmol of 5′-^32^P-labeled AAUAAAUAU derivative in 0.5 μl of buffer A was added to the complexes and to a control mixture containing 9 pmol of vacant 40S subunits. The cross-linking procedure and the subsequent analysis of cross-linked uS3 by polyacrylamide gel electrophoresis (PAGE) in the presence of sodium dodecyl sulfate (SDS) were carried out as described (6,7).

### 40S subunit complexes for EPR measurements

Reactivated subunits were incubated with a doubly spin-labeled oligomer in buffer A for 40 min at room temperature to obtain a binary complex with the 40S ribosomal subunits. Typically, the final concentrations of 40S subunits and the derivative were 15 μM and 13 μM, respectively. If necessary, the 40S subunits were preincubated with 300 μM unmodified undecaribonucleotide UGUGUUCUAAA before adding the RNA derivative. When binding a doubly spin-labeled RNA derivative to 40S subunits complexed with an unmodified short mRNA and P site tRNA, the appropriate ternary complex of 40S subunits was preformed in buffer A as described above. Generally, it was obtained using 100–130 pmol of 40S subunits, the same amount of mRNA and 350–500 pmol of tRNA^Asp^ or tRNA^Phe^ (cognate to the 5′-terminal triplet of the respective mRNA) with a final volume of 5 μl. The occupation of the 40S subunit channel by mRNA in the presence of tRNA cognate to its 5′-terminal triplet was examined in separate experiments with the 5′-^32^P-labeled mRNA using the nitrocellulose filtration technique, as described (17) (the details are given in the Supporting information).

### EPR experiments

DEER distance measurements were performed at Q-band (34 GHz) at a temperature of 50 K with a Bruker Elexsys E580 EPR spectrometer equipped with EN5107D2 resonator and Oxford Instruments temperature control system. Samples of ca. 7 μl volume in quartz tubes (OD 1.65 mm, ID 1.15 mm) were shock frozen prior to insertion into the precooled resonator. A standard four-pulse DEER sequence (18) was applied with 44-56 ns observer π-pulses and 100 ns pump π-pulse and with two-step phase cycle; the available microwave power was 1 W. The time delay *d*_1_ = 340 ns, *d*_2_ = 4000 ns, with a shot repetition time of 5000 μs and time increments of 8 ns. The spin echo was observed at the central line of the nitroxide EPR spectrum and the pump pulse was applied 60 MHz away to the low-field line. Nuclear modulation artifacts arising from matrix deuterons were eliminated by adding traces for eight values of *d*_1_ with increments Δ*d*_1_ = 16 ns. Time-domain dipolar evolution traces were analyzed using DeerAnalysis2016 (19). Distance distributions were characterized in terms of its mean value <*r*_DEER_> and standard deviation σ.

## RESULTS AND DISCUSSION

### Short RNA binds to 40S subunits even prebound to mRNA or tRNA

**Figure 2.**
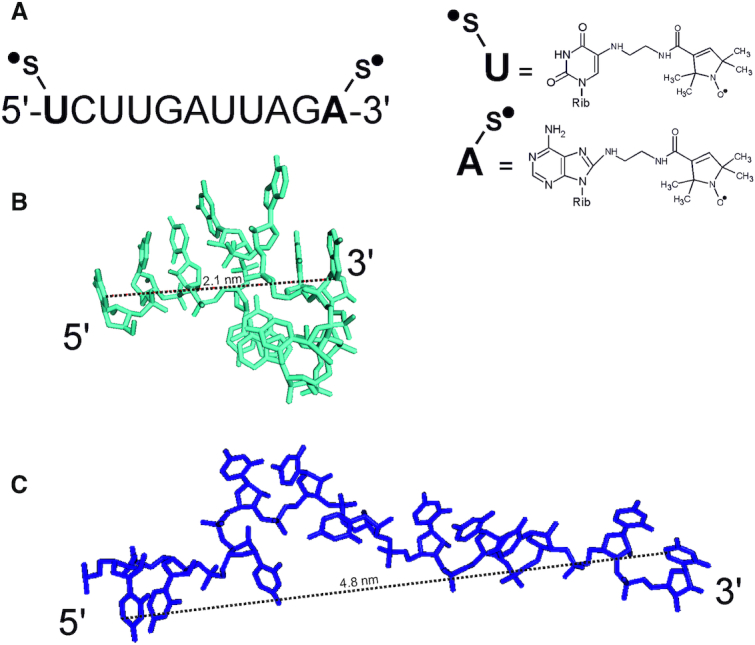
MR11 and its spatial structure. (**A**) The sequence of MR11 and the structures of nucleotides bearing spin labels. (**B**) The predicted 3D stick structure of free MR11 and the calculated distance between nucleotide atoms bearing spin-labeled groups (dashed line). (**C**) The structure of RNA 11-mer in the extended form typical for mRNA stretch bound at the 40S subunit channel (extracted from the structure of the 80S ribosomal complex containing mRNA and tRNAs, PDB ID: 4UJE).

The ability of uS3 to cross-link to various reactive oligonucleotide derivatives is a specific feature of the human 40S ribosomal subunit, both free and when associated with the 60S subunit in the 80S ribosome. This cross-linking is rather efficient even though the affinity of oligonucleotides for the 40S subunit or the 80S ribosome is very low compared with the affinity of mRNA analogues for the 40S ribosomal channel in the presence of a cognate tRNA that fixes them there (3–5). The low affinity makes it difficult to detect the formation of ribosomal binary complexes with oligonucleotides using standard biochemical methods. Therefore, we used our previous approach (9), to study some features of the 40S subunit binary complex with short RNA. This approach can detect conformational changes in double spin-labeled RNA caused by its binding to 40S subunits, and readily reveals them through changes in their <*r*_DEER_> values. The distance between the terminal nucleotides in MR11 is different depending on its state, whether free in solution or bound in binary complexes with the 40S subunit. The MR11 derivative is an RNA 11-mer with one spin label at the 5′-terminal uridine and another at the 3′-terminal adenosine (Figure 2A). According to the 3D structural prediction, free MR11 folds into a compact structure, where the distance between the nucleotide atoms bearing the spin label groups is about 2.1 nm (Figure 2B), while the extended form of MR11 should be similar to that of mRNA stretch bound at the 40S subunit channel (Figure 2C).

**Figure 3.**
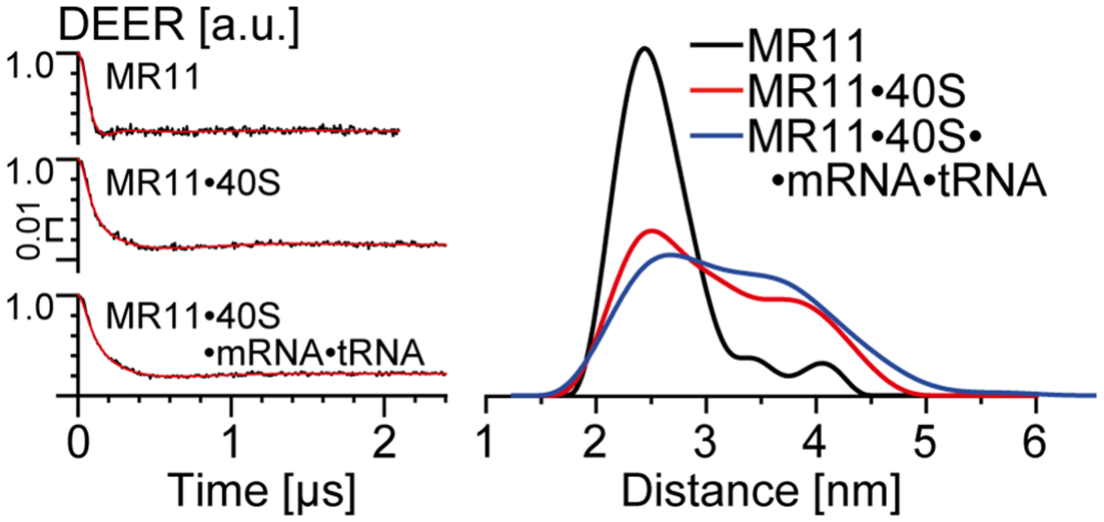
Distance measurements obtained by DEER for isolated MR11, its binary mixture with 40S subunits and its mixture with the ternary complex of 40S subunits with 19-mer mRNA GACUUCAACAAACACAACU and P site tRNA^Asp^. (Left) Background corrected four-pulse DEER traces (intensity is normalized). Lines with noise correspond to the experimental data. Solid lines show best fits obtained using DeerAnalysis2016. (Right) Obtained distance distributions. Note that distance distribution in the presence of 40S subunits is quite broad and probably contains two unresolved peaks from the unfolded and the compact MR11 forms.

We expected to obtain <r_DEER_> values for different MR11 conformations, including one specific for its binary complex with the 40S subunit, similar to an analogous RNA 11-mer derivative (9), because, according to our data on the cross-linking of RNA derivatives to uS3 mentioned in the Introduction, this type of complexes should not depend on the RNA oligomer sequence. Specifically, the free MR11 (compact monomer) and the MR11 bound in the binary complex with the 40S subunits, would have <*r*_DEER_> values of 2.1 and 3.6 nm, respectively. The distance distributions in Figure 3 show indeed that for an isolated MR11, the <r_DEER_> value is 2.5 nm, meaning that in this state it is mostly in the compact monomer form. The obtained value differs somewhat from 2.1 nm, since the latter is the distance calculated between the 5′- and 3′-terminal bases in the unmodified oligomer, but not between spin labels attached to these bases via chemical linkers. However, when MR11 was mixed with 40S subunits, this value increased to 3.3 nm, which indicates the unfolding of the compact form, accompanying the binding of MR11 to the 40S subunit, similar to what has been observed in our previous study (9).

Remarkably, when the free 40S subunits were replaced with their complex with the mRNA 19-mer GACUUCAACAAACACAACU fixed in the mRNA binding channel by the interaction of its 5′-terminal triplet GAC with the cognate tRNA^Asp^ in the P-site, no changes in the distance distribution were observed (Figure 3 and Supplementary Figures S7, S9). This mRNA is long enough to occupy the entire channel from the P site up to the entry site, which accommodates 19 nucleotides (e.g., see 20 and 21). The almost complete filling of the channel by this RNA oligomer was confirmed by binding of ^32^P-labeled mRNA to the corresponding 40S subunit sample in the presence of cognate tRNA^Asp^ (see Supporting information). Consequently, the binding of MR11 to 40S subunits must occur outside the mRNA binding channel. These results are in good agreement with earlier data that cross-links of dialdehyde oligoribonucleotide derivatives to uS3 do not depend on the occupation of the mRNA binding channel with short mRNA analogues (6).

### The putative RNA binding site in 40S subunits lies between the mRNA entry site and K62 of uS3

**Figure 4.**
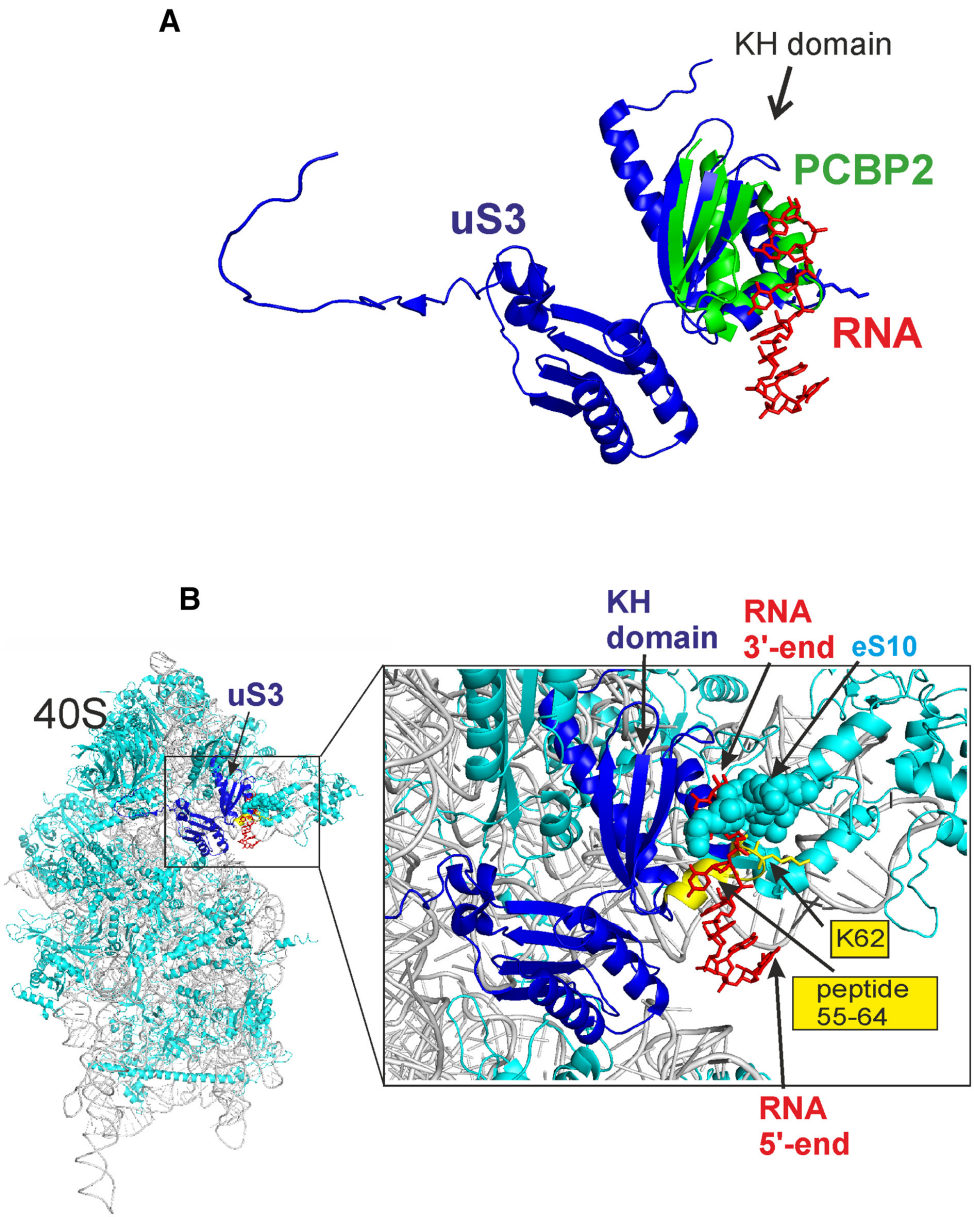
Modeling of the location of an RNA stretch in its potential binding site on the uS3 KH domain. (**A**) The KH domain of human PCBP2 (green) complexed with an RNA (red) (PDB ID: 2PY9 (22)) docked into the KH domain of uS3 (dark blue) extracted from the human 40S subunit (PDB ID: 4V6X (23)). The docking was performed by the pair fitting utility of Pymol software (24) using atom coordinates of the GXXG loop that is conserved in all KH domains. (**B**) The same RNA stretch is displayed on the model of the 40S ribosomal subunit. The peptide 55–64 of uS3 and its K62 are shown in yellow; the C-terminal tail of ribosomal protein eS10 is marked as cyan spheres.

The so-called ‘KH domain’ in uS3 is an obvious candidate for the site where labile binding of unstructured RNAs occurs, resulting in cross-linking of 3′-dialdehyde derivatives to the uS3 fragment 55–64. This domain is a well-known conserved structural fold responsible for the binding of single-stranded RNAs and DNAs peculiar to a number of proteins. There is a high degree of structural similarity with other members of the KH domain family of proteins, whose complexes with RNA have been studied by X-ray crystallography. This makes it possible to model the location of an oligoribonucleotide in a binary complex with the 40S subunit. For example, the KH domain of human poly(C)-binding protein-2 (PCBP2) (22) is easily aligned with the KH domain of uS3 in the 40S subunit (Figure 4A), and the four nucleotide-long RNA stretch bound to the KH domain of PCBP2 provides a plausible structure for an RNA fragment bound to the uS3 KH domain (Figure 4B). A close examination of the resulting model for RNA in the KH domain of uS3 (Figure 4B) shows that this pocket indeed contains peptide 55–64, but is not readily available for binding of RNAs because the ribosomal protein eS10 tail partially shields it. Moreover, if a four-nucleotide-long RNA fragment was bound to this hypothetical uS3 pocket, its 3′-terminal ribose would be oriented away from peptide 55–64 and its K62 (6), rather than in their immediate vicinity (Figure 4B). Thus, the expected placement of unstructured RNA in its uS3 KH domain binding pocket is unfavorable for the cross-linking of 3′-dialdehyde RNA derivatives to K62, which suggests that RNA is arranged differently in its binary complex with the 40S subunit. In addition, only a short portion of the RNA 11-mer would participate in binding to the 40S subunit. Thus, the labile binding of unstructured RNAs on the 40S subunit likely occurs elsewhere.

**Figure 5.**
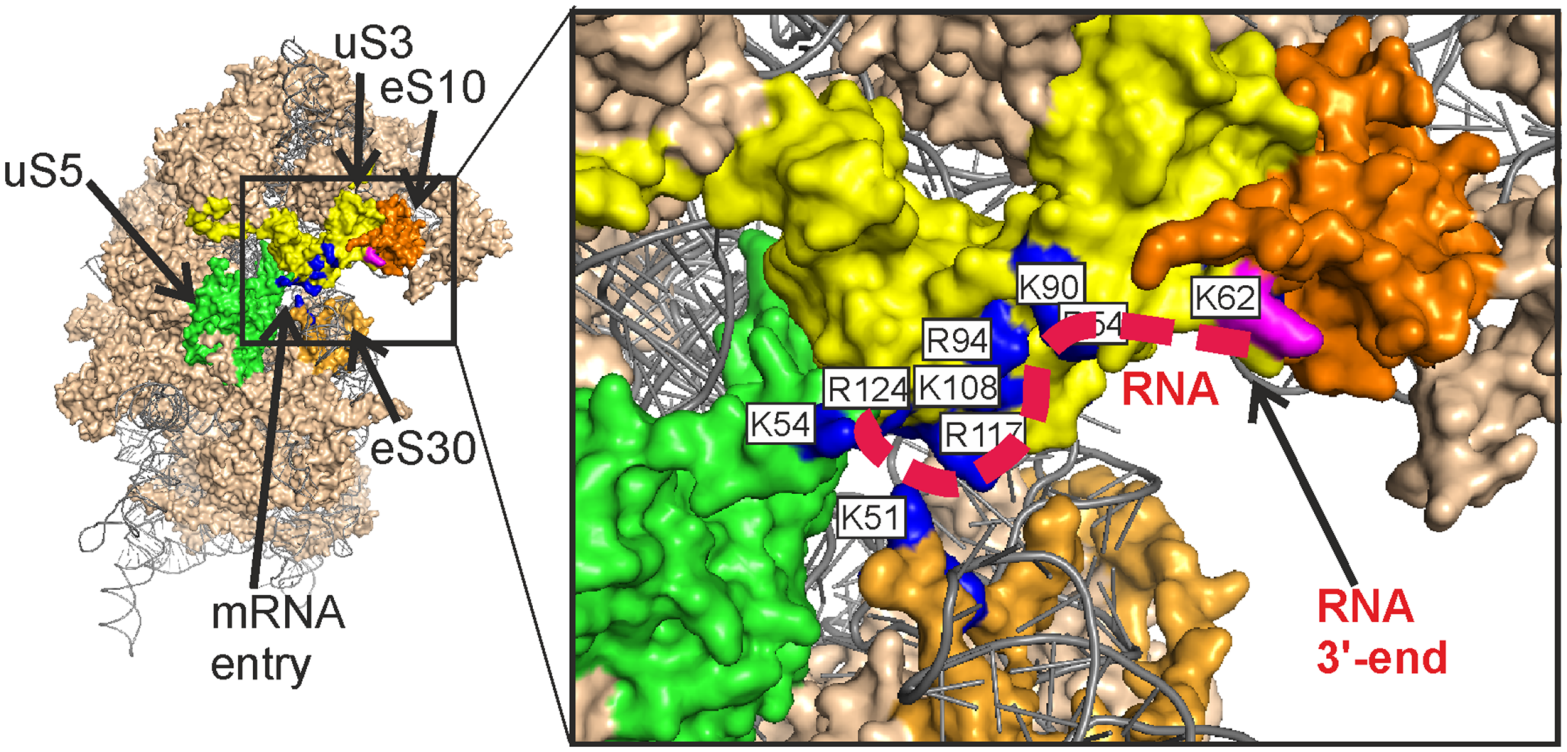
The proposed binding site for unstructured RNAs near the mRNA entry site of the 40S ribosomal subunit. Solvent side view of the 40S subunit (PDB ID: 4V6X (23)). Ribosomal proteins are shown in the ‘surface’ view. The positively charged amino acid residues of the ribosomal proteins uS3 (yellow), uS5 (green) and eS30 (light orange), which could form a binding pocket for unstructured RNAs, are shown in dark blue and labeled with white rectangles. The predicted location of an RNA 11-mer is shown with a red dashed line. The residue K62 in uS3 is colored by magenta. Ribosomal protein eS10 is shown in orange.

Where could RNA binding occur if not in the uS3 KH domain? Careful inspection of the environment of uS3 peptide 55–64 reveals an exposed pocket on the 40S subunit surface for binding of short RNA. The 3′-terminal ribose of the RNA when placed in this pocket could easily contact peptide 55–64. There is a cluster of positively charged amino acid side chains in the uS3 C domain, which forms the above pocket, along with the neighboring K54 from uS5 and K51 from eS30, which could be involved in the binding of a short single-stranded RNA (Figure 5). If the 5′-terminal part of such RNA is bound to this pocket, its 3′-terminal ribose has ready access to K62. This region of the 40S subunit between the mRNA entry site and K62 of uS3 is the strongest candidate for the site of labile binding of unstructured RNAs and is able to accommodate an 11-mer.

### Labile binding of RNA is affected by mRNA in its channel

**Figure 6.**
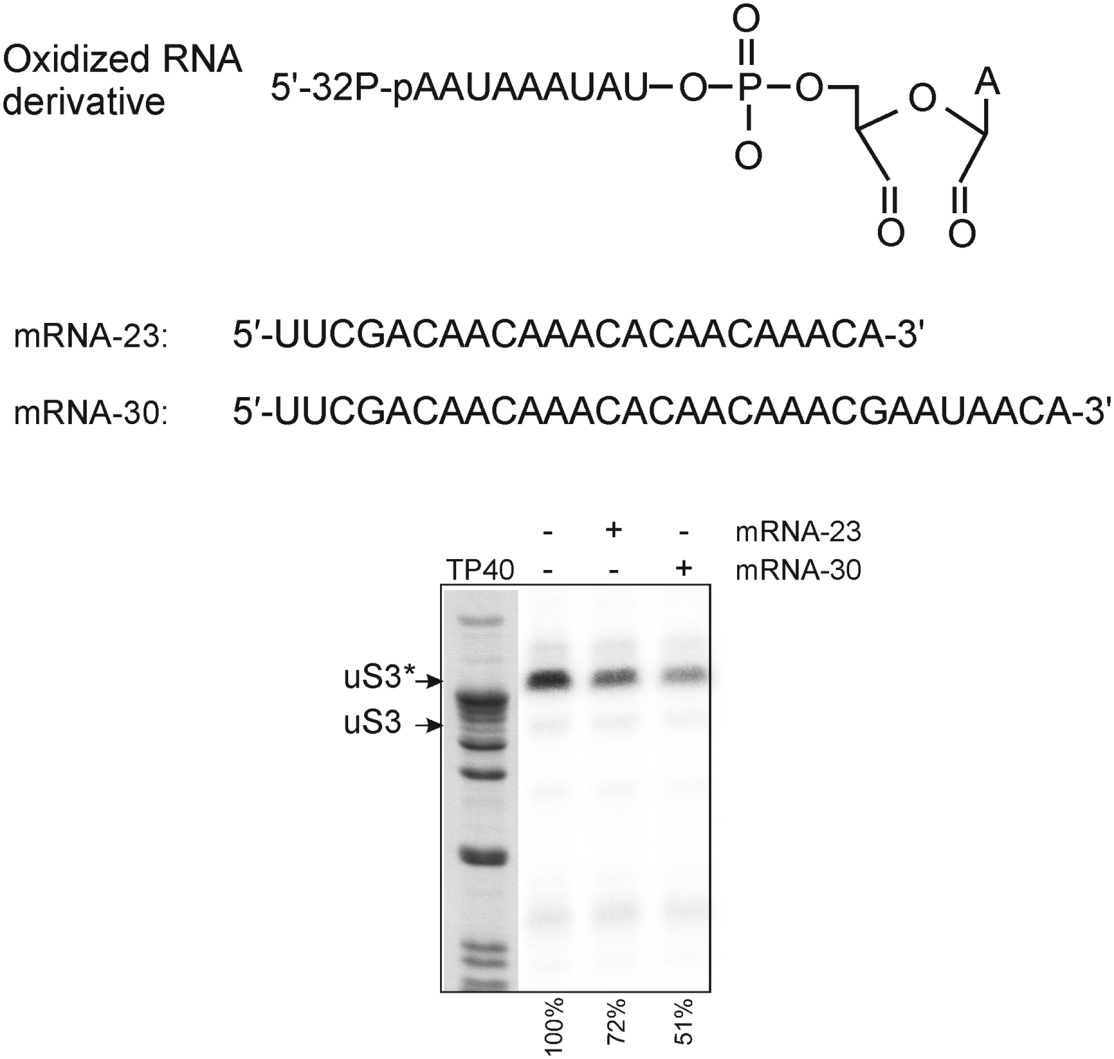
The effect of binding of 23-mer or 30-mer mRNA in the ribosome channel on the cross-linking of the 5′-^32^P-labeled 3′-dialdehyde derivative of nonaribonucleotide AAUAAAUAU to uS3 (mRNAs were fixed on the 40S subunits by tRNA^Phe^ cognate to their 5′-terminal UUC triplets). The autoradiogram of the gel after the separation of ribosomal proteins isolated from the 40S subunits cross-linked in the presence and absence of mRNAs (shown above the electrophoretogram). TP40, a stained lane of total protein of the 40S ribosomal subunit, where the position of uS3 is shown according to western blot data (see Figure 7). Bands of cross-linked uS3 are marked as uS3*. Quantification of the results is given below the autoradiogram relative to uS3 cross-linked without mRNAs.

Remarkably, the uS3 fragment 55–64 can cross-link to mRNA fixed in the mRNA binding channel via apurinic/apyrimidinic (abasic) site in the 3′-terminal tail hanging outside the ribosome (25). It follows that unmodified 3′-terminal fragments of mRNAs bound in the channel, which protrude outside the ribosome, should interfere with the cross-linking of a 3′-dialdehyde oligoribonucleotide derivative to the uS3 fragment 55–64. We tested this hypothesis with 23-mer and 30-mer mRNA analogues similar to those used in (25) but without abasic sites (Figure 6). These mRNAs were fixed in the channel of the 40S subunit by the interaction of their 5′-terminal UUC triplets with the P site tRNA^Phe^. Nucleotides located downstream of the position +19 (counting from the first nucleotide of the P-site codon) are outside the ribosome (e.g. see 20 and 21) and protrude from the mRNA entry site.

Figure 6 demonstrates that mRNAs in the 40S subunit channel indeed decrease the yield of cross-links of the 3′-dialdehyde nonamer RNA derivative to the uS3 peptide 55–64 and this effect is more pronounced for the 30-mer mRNA with the longer hanging fragment. Thus, mRNA fragments protruding from the mRNA entry site interfere with the binding of the oligoribonucleotide derivative to the 40S subunit and thereby impede its cross-linking to the uS3 peptide. Because abasic sites located at the 3′-termini of the mRNAs used can cross-link to uS3 peptide 55–64 (25), we conclude that the oligoribonucleotide binding site lies in the area between the mRNA entry site and the K62 of uS3. This region coincides with the cluster of positively-charged amino acid residues of the ribosomal proteins uS3, uS5 and eS30 (Figure 5) proposed above as the site for labile binding of unstructured RNAs on the 40S subunit. We also attempted to detect a similar effect from mRNA protruding from the mRNA entry site on the binding of MR11 to the 40S subunit. The portion of the 30-mer mRNA hanging outside the 40S subunit channel impedes binding of MR11 to a much lesser extent than it affects cross-linking to uS3 (Supplementary Figure S4), suggesting that the nitroxide spin labels contribute to the affinity of MR11 to the 40S subunit.

### The 3′-terminus of a loosely-bound RNA 11-mer is about 4 nm from the mRNA entry site

We examined the intermolecular spin-spin interaction between labels attached to the 3′-terminal nucleotides of mRNA and loosely bound RNA to find the distance between the ribosome channel and bound unstructured RNA. We used the 19-mer mRNA GACUUCAACAAACACAACU spin-labeled at the 3′-terminal U. The label sits at the mRNA entry site when the mRNA is fixed in the 40S subunit channel using the codon-anticodon interaction between its 5′-terminal triplet GAC and the P site tRNA^Asp^. We also utilized derivatives of a nonaribonucleotide AAUAAAUAU (whose 3′-dialdehyde derivative was used for cross-linking to the uS3 peptide 55–64) with a spin label at the 5′- or 3′-terminal nucleotide. Only with the 3′-nonamer derivative could we detect a weak DEER signal (Supplementary Figure S5) showing a broad distribution of spin-spin distances with <*r*_DEER_> = 3.6, corresponding to the proposed position of the labile bound RNA in the region between the mRNA entry site and K62 of uS3 (Figure 5). The large width of the <*r*_DEER_> distribution shows a large variability of positioning of the loosely bound nonaribonucleotide derivative on the 40S subunit. The signal is weak because of the small amount of spin-spin pairs, resulting from the low level of binding of the nonamer derivative to the 40S subunits. In the previous section, we suggested that spin labels enhance the affinity of doubly-labeled RNA oligomer to the 40S subunit due to additional hydrophobic interactions. The enhancing effect of a single spin label seems much weaker than that of two and reduces the level of binding of the labeled RNA derivatives compared with the level of the doubly-labeled RNA. Although the DEER signal is weak, it indeed corresponds to a pair of labels attached to the 3′-terminal nucleotide of the mRNA fixed in the channel and to the oligoribonucleotide derivative bound to the same 40S subunit. First, the 40S subunits are in slight excess over the mRNA, so that the mRNA is completely bound in the 40S subunit channel and none remains outside the ribosome (see Supporting information), eliminating the possibility of interaction between the mRNA and nonaribonucleotide derivative outside the 40S subunit. Second, when the nonamer derivative was replaced by the same 3′-spin-labeled derivative of the tetramer AUCU, no DEER signal was detected (data not shown). These results show that a 4-mer RNA is too short to bind to the proposed pocket of the 40S subunit (Figure 5) even in a labile manner and that in the absence of binding, there is no DEER signal.

### The ribose 2′-OH group contributes to the binding of RNA to 40S subunits

**Figure 7.**
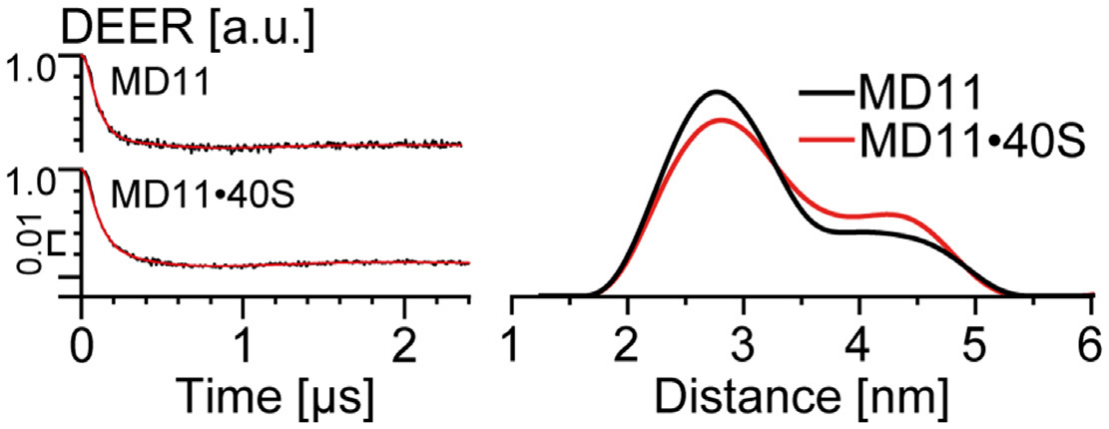
Distance measurements in MD11 and its binary mixture with 40S subunits, obtained by DEER. (Left) Background corrected four-pulse DEER traces (intensity is normalized). Lines with noise correspond to the experimental data. Solid lines show the best fits obtained using DeerAnalysis2016. (Right) The distance distributions. Differences in the distance distribution are the result of experimental noise (see SI, Supplementary Figure S6).

Oligodeoxyribonucleotide derivatives are also able to cross-link to uS3 in binary complexes with 80S ribosomes (5). In addition, single-stranded DNA oligomers containing an abasic site can cross-link to the same uS3 peptide 55–64 as do RNA derivatives with 3′-terminal dialdehyde groups (26). This prompted us to check whether the MD11, a doubly spin-labeled DNA oligomer with a sequence corresponding to that of MR11, can also form a binary complex with the 40S subunit. The results in Figure 7 show that, like MR11, isolated MD11 exists preferentially in the compact monomer form with a major peak at <*r*_DEER_> = 2.9 nm, although a minor peak at <*r*_DEER_> = 4.4 nm, which obviously corresponds to the extended form, is also clearly visible. Unexpectedly, addition of 40S subunits does not significantly change the distribution of distances (Figure 7) and does not increase the fraction of the extended DNA form, as it did for MR11. Thus, complexes of 40S subunits with DNA oligomers are even more labile than binary complexes with RNA oligomers. Much higher concentrations of 40S subunits and oligodeoxyribonucleotides may be needed to detect such complexes, which is difficult to achieve and not physiologically relevant. In general, the data suggest that the 2′-OH groups of the ribose-phosphate backbone of RNA are significant in the formation of its binary complex with the 40S subunit.

Why might the cluster of positively charged amino acid residues between the mRNA entry site and K62 of uS3 prefer to bind RNA rather than single-stranded DNA? It seems unlikely that the 2′-OH groups contribute significantly to the interaction of nucleic acids with positively charged amino acid side chains. However, it is known that such short RNAs and DNAs preferably adopt A and B forms, respectively (e.g. see 27). Possibly, the RNA is a better fit to the configuration of positively charged amino acid residues in the nucleic acid binding pocket on the 40S subunit (Figure 5).

### The binding of RNA to 40S subunits does not involve uS3 peptide 55–64

**Figure 8.**
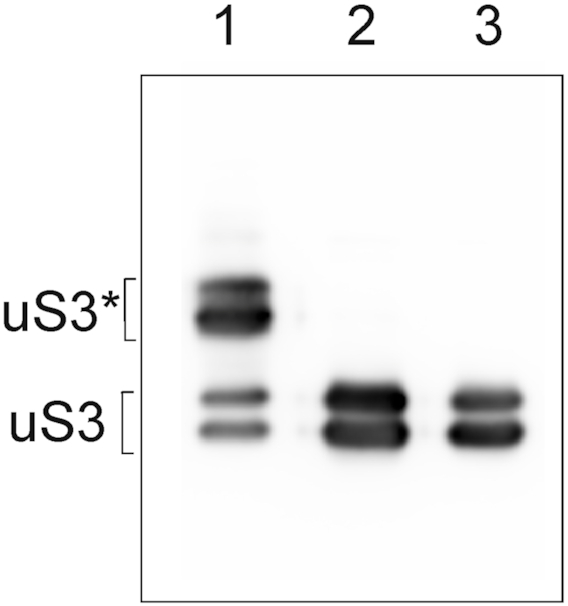
The extent of uS3 cross-linking to the 3′-dialdehyde derivative of nonaribonucleotide AAUAAAUAU in the 40S subunits after purification by pelleting using high speed centrifugation. Western-blot analysis of ribosomal proteins isolated from the subunits treated with the 3′-dialdehyde derivative of AAUAAAUAU (1), with the unmodified 9-mer (2), and without oligonucleotides (3) using specific antibodies against mammalian uS3. The bands corresponding to cross-linked uS3 are marked with asterisk. Note that the uS3 protein isolated from human placental 40S subunits migrates in SDS-PAGE as two bands corresponding to its phosphorylated and unphosphorylated forms (6). The doubling of the uS3 bands is barely visible in Figure 6 because of the shorter run used there.

The exposed peptide 55–64 of uS3 is a target for cross-linking of oligoribonucleotide derivatives (6), it seems natural that labile binary complexes of short unstructured RNAs with 40S subunits are formed with the participation of this peptide. However, cross-linking may occur because other interactions position the short oligonucleotides near uS3 peptide 55–64. To clarify this issue, we examined MR11 binding to 40S subunits, in which the uS3 was already cross-linked to an oligoribonucleotide derivative lacking a spin label. An oligoribonucleotide with 3′-ribose oxidized to dialdehyde gives a high yield of cross-linking to the uS3 peptide in 40S subunits (6). Western-blot analysis of 40S subunits treated with the 3′-dialdehyde derivative of the AAUAAAUAU 9-mer showed that ∼60% of the 40S subunits contained uS3 cross-linked to the RNA 9-mer (Figure 8). Thus, if the uS3 peptide 55–64 is crucial for binding oligoribonucleotides, these 40S subunits with the cross-linked uS3 should be incapable of binding. In this case, with 60% of 40S subunits cross-linked in our sample, the size of the DEER peak corresponding to the binary complex of 40S subunits with MR11 would be reduced by about half and the narrow peak at 2 nm corresponding to compact monomer form would be prominent.

**Figure 9.**
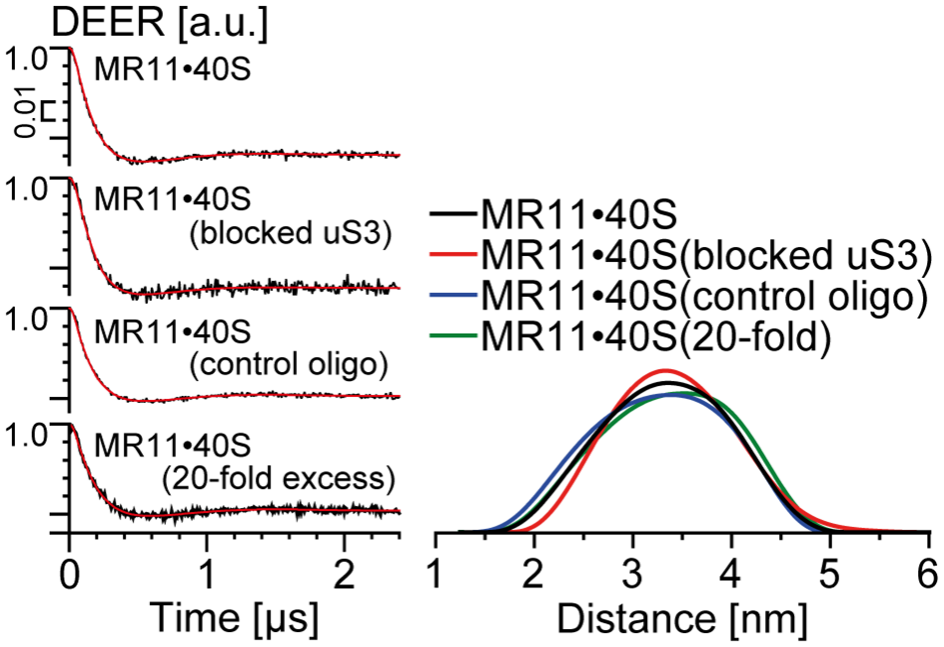
Distance measurements by DEER in binary mixture of MR11 with cross-linked 40S subunits, with 40S subunits subjected to the same treatments as cross-linked without any additional components and in the presence of unmodified nonaribonucleotide AAUAAAUAU or the 20-fold excess of the unmodified 11-mer UGUGUUCUAAA. (Left) Background corrected four-pulse DEER traces (intensity is normalized). Lines with noise correspond to the experimental data. Solid lines show best fits obtained using DeerAnalysis2016. (Right) The obtained distance distributions. Differences in the distance distribution are the result of experimental noise (see SI, Supplementary Figures S8 and S9).

Figure 9 shows that the distance distributions from DEER for MR11 with cross-linked 40S subunits are very similar to those from control experiments with 40S subunits subjected to the same treatments but either with unmodified nonaribonucleotide AAUAAAUAU or without added RNA. The distance distributions display the broad peak, characteristic of bound MR11 in Figure 3, but without the sharp peak near 2 nm, characteristic of free, compact MR11. There is no indication that more than half of the 40S subunits in the cross-linked sample are not capable of binding MR11. It follows that the blocking of uS3 peptide 55–64 has no noticeable effect on the formation of a binary complex of the spin-labeled oligoribonucleotide with 40S subunits. This means that uS3 peptide 55–64 is not involved itself in the binding interactions of uS3 with RNAs, even though it is the target for cross-linking reactions with oligonucleotide derivatives. Thus, binding occurs near but independently of uS3 peptide 55–64. But binding does position the RNA close enough to uS3 peptide 55–64 for efficient cross-linking to occur.

It should be noted that the unmodified 9-mer, the 11-mer UGUGUUCUAAA and the hanging portion of 30-mer mRNA are all poor competitors for MR11 in its binding to the 40S subunits. Even a 20-fold excess of the unmodified 11-mer did not cause a significant decrease in the amount of MR11 bound in the binary complex, as can be seen from the respective distance distributions in Figure 9. These results support the suggestion above that the nitroxide spin labels on the terminal nucleotide bases of MR11 enhance its affinity for the 40S subunit due to additional hydrophobic interactions provided by these labels. These additional interactions probably allowed us to detect the binary complex of MR11 with the 40S subunit using EPR spectroscopy, while the binding of other types of oligoribonucleotide derivatives to the free 40S subunits can barely be detected by conventional nitrocellulose filtration techniques ((3–5); Supplementary Figure S1). It is necessary to note that small shift of the binding equilibrium upon shock-freezing compared to room temperature hypothetically might take place.

## CONCLUSION

This study shows that labile binding of unstructured RNAs to human 40S ribosomal subunits occurs away from the mRNA channel and takes place in the region between the mRNA entry site and K62 of uS3. The RNA binding pocket is formed by a cluster of positively charged amino acid residues of ribosomal proteins, mainly of uS3 and a few of eS5 and eS30. DEER/PELDOR spectroscopy revealed a number of quite unexpected details about the formation of complexes of 40S subunits with unstructured RNAs. In particular, the uS3 peptide 55–64 in the single stranded nucleic acid-binding KH domain does not participate in the binding even though cross-linking of oligonucleotide derivatives to this uS3 peptide 55–64 is the main biochemical evidence for such complexes. In addition, we found that the 40S subunit strongly prefers to bind unstructured RNA rather than single-stranded DNA, although short DNA strands can cross-link to the uS3 peptide 55–64 in the 40S subunit via an abasic site.

The location of the binding pocket for unstructured RNA and the fact that the binding is not affected by filling the 40S subunit channel with mRNA suggest some functional implications for the labile binding of unstructured RNAs. The site shows no strong sequence specificity, so it seems unlikely that this site actually would function by interacting with RNAs other than mRNAs. Most likely, the role for this binding site is to bind segments of mRNAs queued up at the entry site during translation. This role explains why several amino acid residues of the proposed binding pocket, namely R117 and K108 of uS3, K54 of uS5 and K51 of eS30, are located very close to the mRNA in the 48S preinitiation complex (28), where they would directly interact with mRNA during translation initiation. The R116/R117 of uS3 are key players in stabilizing the contacts of mRNA in the entry site region, which is important for proper recognition of the start codon by the 43S preinitiation complex upon mRNA scanning (29). Thus, one functional assignment for the cluster of positively charged amino acid residues in the region of the mRNA entry site may be to attract mRNAs to this region and facilitate their entry into the 40S subunit channel. During translation elongation, the 40S subunit area between the mRNA entry site and the uS3 peptide 55–64 is positioned to play a role in mRNA quality control. The K62 of this peptide can form cross-links with aldehyde groups in unstructured RNAs and might also interact with the open aldehyde form of abasic sites (30), which arise from exposure of cells to a variety of endogenous and exogenous factors (31). The ability of ribosome-bound mRNA containing an abasic site to cross-link to the 40S subunit via uS3 peptide 55–64 has recently been demonstrated (25). The cluster of positively charged amino acid residues between the K62 of uS3 and the mRNA entry site likely facilitates the inspection of mRNA before it enters the 40S subunit channel so that K62 can form a cross-link with any abasic site in the mRNA. Obviously, the binding of mRNA just to this cluster, which was modeled here by the complexation of unstructured RNAs to the 40S subunit outside the mRNA channel, should be weak and labile so as not to prevent the movement of the mRNA through the ribosome in the course of translation. The next stage in this research is to obtain direct confirmation that the 40S subunit area suggested here as the putative site for labile binding of unstructured RNAs does interact with mRNAs during translation.

## Supplementary Material

gkz1039_Supplemental_File
