# Supplementary material for: Exploring the interactions of short RNAs with the human 40S ribosomal subunit near the mRNA entry site by EPR spectroscopy
Source: Nucleic Acids Res. 2019 Nov 14;47(22):11850–60. doi: 10.1093/nar/gkz1039 (PMC7145563; doi:10.1093/nar/gkz1039)
Supplement: gkz1039_Supplemental_File [file gkz1039_supplemental_file.pdf]

SUPPORTING INFORMATION

Exploring the interactions of short RNAs with the human 40S ribosomal subunit near the mRNA entry site by EPR spectroscopy

Alexey A. Malygin,[a,c,d] , Olesya A. Krumkacheva,[b,c,d] Dmitri M. Graifer,[a,c,d] , Ivan O. Timofeev, [b,c,d] Anastasia S. Ochkasova,[a,c] Maria I. Meschaninova,[a,c] Alia G. Venyaminova,[a,c] Matvey V. Fedin,[*b,d] Michael Bowman,[c,e] Galina G. Karpova[*a,c,d] and Elena G. Bagryanskaya[*c,d]

[a]*Institute of Chemical Biology and Fundamental Medicine SB RAS, Pr. Lavrentjeva 8, Novosibirsk, 630090, Russia;*

[b]*International Tomography Center SB RAS, Institutskaya Str. 3a, Novosibirsk, 630090, Russia;*

[c]*N. N. Vorozhtsov Novosibirsk Institute of Organic Chemistry SB RAS, Pr. Lavrentjeva 9, Novosibirsk, 630090, Russia;*

[d]*Novosibirsk State University, Pirogova Str. 2, Novosibirsk, 630090, Russia*

[e]*University of Alabama, Tuscaloosa, Alabama 35487-0336, United States*

Table of contents

Estimation of the extent of the ternary complex formation between human 40S subunits, P site tRNA and an mRNA analogue 2

CW EPR study of nitroxide labeled oligonucleotides 3

DEER distance measurements 6

Raw traces and validation of DEER data 7

Estimation of the extent of the ternary complex formation between human 40S subunits, P site tRNA and an mRNA analogue

It is difficult to measure directly the extent of binding of mRNA analogue at the 40S ribosomal channel under conditions used for the EPR experiments because the subunits concentration is too large for carrying out a conventional approach utilizing examination of the amount of ribosomes bound to a labeled ligand by nitrocellulose filtration technique. Therefore, we used this technique to obtain binding isotherms for the labeled mRNA analogue at much lower concentrations of the analogues and 40S subunits to estimate lower limit of the level of mRNA analogues binding to 40S subunits in the presence of a $tRNA^{Asp}$ or $tRNA^{Phe}$ cognate to the 5'-terminal mRNA triplet GAC or UUC, respectively. We examined binding properties of the mRNA analogues on the example of 19-mer GACUUCAACAAACACAACU and 30-mer UUCGACAACAAACACAACAAACGAAUAACA.

Binding experiments were carried out with the same 40S preparation as EPR experiments. Prior to these experiments, the mRNA analogue was labeled at the 5'-terminus by ^{32}P with the application of polynucleotide kinase and [γ-^{32}P]ATP; the labeled nonamer was purified by HPLC. Binding of the ^{32}P-labeled mRNA analogues to 40S ribosomal subunits was examined in buffer A at 25°C by standard nitrocellulose filtration technique with the use of 0.45 μm pore size filters pretreated with 0.6 M KOH at 25°C for 15 min in order to decrease level of unspecific sorption of free RNA. Concentrations of the labeled mRNA analogues in the binding assay mixtures was 0.3 μM; concentration of 40S subunits varied from 0.02 μM to 0.8 μM, tRNA (where used) was taken in a 5–fold excess relative to the subunits. Each binding mixture contained 3 pmol of the labeled 23- or 30-mer and the respective amounts of other components.

The results presented in Figure S1 show that binding of the labeled mRNA analogue is almost completely dependent on the presence of tRNA cognate to its desired 5'-terminal triplet and is barely detectable without tRNA; at the equimolar ratio (0.3 μM 40S subunits), < 96 % of the mRNA analogue is tightly bound at the ternary complex with 40S subunits.

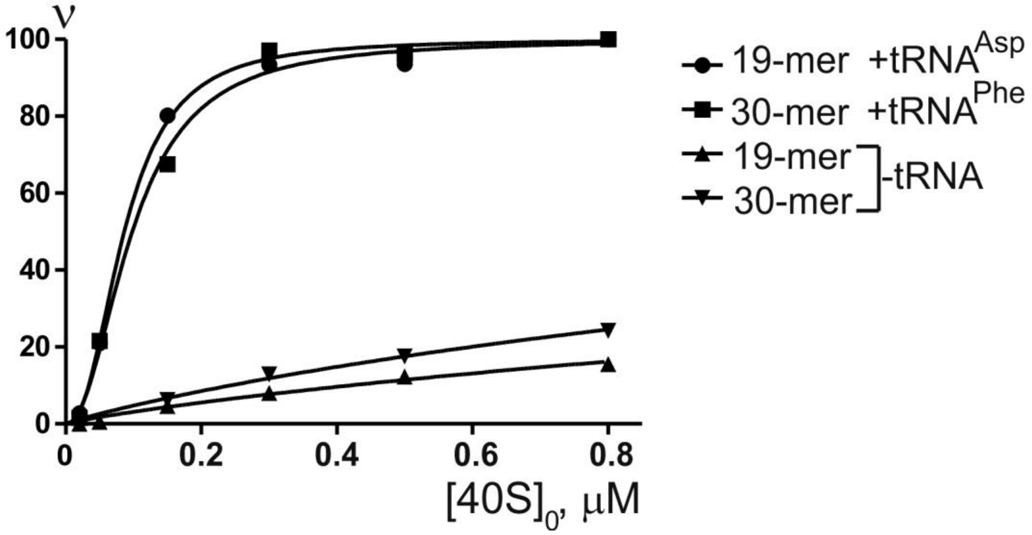

Figure S1. Binding of 5'-^{32}P-labeled RNA 19-mer GACUUCAACAAACACAACU or 30-mer UUCGACAACAAACACAACAAACAGAAUAACA (taken at a concentration of 0.3 μM) to 40S subunits in the presence of $tRNA^{Asp}$ or $tRNA^{Phe}$, respectively (upper curves), and without tRNA (lower curves). ν, binding level (mol of mRNA analogue, per cent of 40S subunits containing the bound oligomer).

CW EPR study of nitroxide labeled oligonucleotides

Continuous wave (CW) EPR spectroscopy at 300K was used to determine radicals concentration and to verify their covalent attachment to oligonucleotides. CW EPR experiments were carried out at X-band (9 GHz) at 300 K using a commercial Bruker EMX spectrometer. Experimental settings were as follows: sweep width 6 mT; microwave power 20 μW; modulation frequency 100 kHz; modulation amplitude 0.05 mT; time constant 81.92 ms; number of points 1024; number of scans - 32. All spectra were simulated using EasySpin [www.easyspin.org] software and slow-motion regime to describe label mobility.

Table S1 shows concentrations of spin labels obtained by CW EPR and concentrations of oligonucleotides determined by the optical absorption assuming that one unit of optical density at 260 nm corresponds to 8600 pmol of double spin-labeled MR11 or MD11, 9400 pmol of spin-labeled 9-mers and 6200 pmol of spin-labeled 19-mer. The spin labels content in MR11 was examined by EPR and, typically, it differed no more than 40% from that determined by the optical absorption of double spin-labeled RNA at 260 nm, assuming that one A_{260} unit of the undecamer corresponds to 8600 pmol (9). There is a good agreement between concentration of oligonucleotides

and corresponding concentration of labels indicating that spin-labeling procedures were performed successfully.

Table S1. The concentrations of oligonucleotides (C_{olig}) determined by the optical absorption and concentrations of spin labels (C_{NIT}) obtained by CW EPR.

Sample	$C_{olig}\times10^5$, M/L	$C_{NIT}\times10^5$, M/L
doubly spin-labeled MR11	5.4	9.5
doubly spin-labeled MD11	2.9	6
singly spin-labeled 9-mer(3') (AAUAAAUAU-NIT)	9	9
singly spin-labeled 9-mer(5') (NIT-pAAUAAAUAU)	9	9
singly spin-labeled 19-mer (GACUUCAACAAACACAACU-NIT)	3.2	3.5

CW EPR spectrum of MR11 (Figure S2) was simulated assuming contributions of two types of spin labels with different rotational correlation times and g=[g_{xx} 2.0061 2.0022] and A= [0.58 0.58 A_{zz}] mT (the values of g_{xx} and A_{zz} were varied). The main fraction of the spectrum (90%) has t_{corr} = 0.46 ns, g_{xx} = 2.0083, A_{zz} = 3.63 mT, and the rest of spin labels (10 %) has t_{corr} =0.08 ns, g_{xx} = 2.0090, A_{zz} = 3.69 mT.

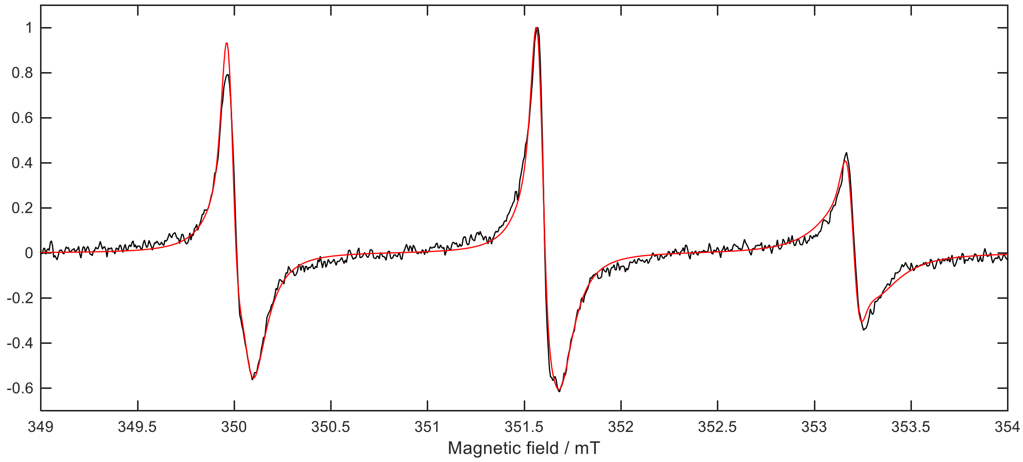

Figure S2. Room-temperature X-band CW EPR spectrum of MR11 in water. Black line – experimental, red line – simulated data.

EPR spectra (Fig. S3) of MD11, 3-mer and 19-mer oligonucleotides consist of one component with t_{corr} and A_{zz} listed in Tables S2.

Table S2. The parameters of simulations shown in Fig. S4. For spin-labeled MR we used g=[2.0083 2.0061 2.0022] and A= [0.58 0.58 A_{zz}] mT in all simulations; the values of A_{zz} were varied.

Sample	t_{corr} / ns	A_{zz} / mT
MDII	0.13	3.67
9-mer(3')<br>AAUAAAUAU-NIT	0.17	3.68
9-mer(5')<br>NIT-pAAUAAAUAU	0.10	3.68
19-mer<br>GACUUCAACAAACACAACU-NIT	0.22	3.67

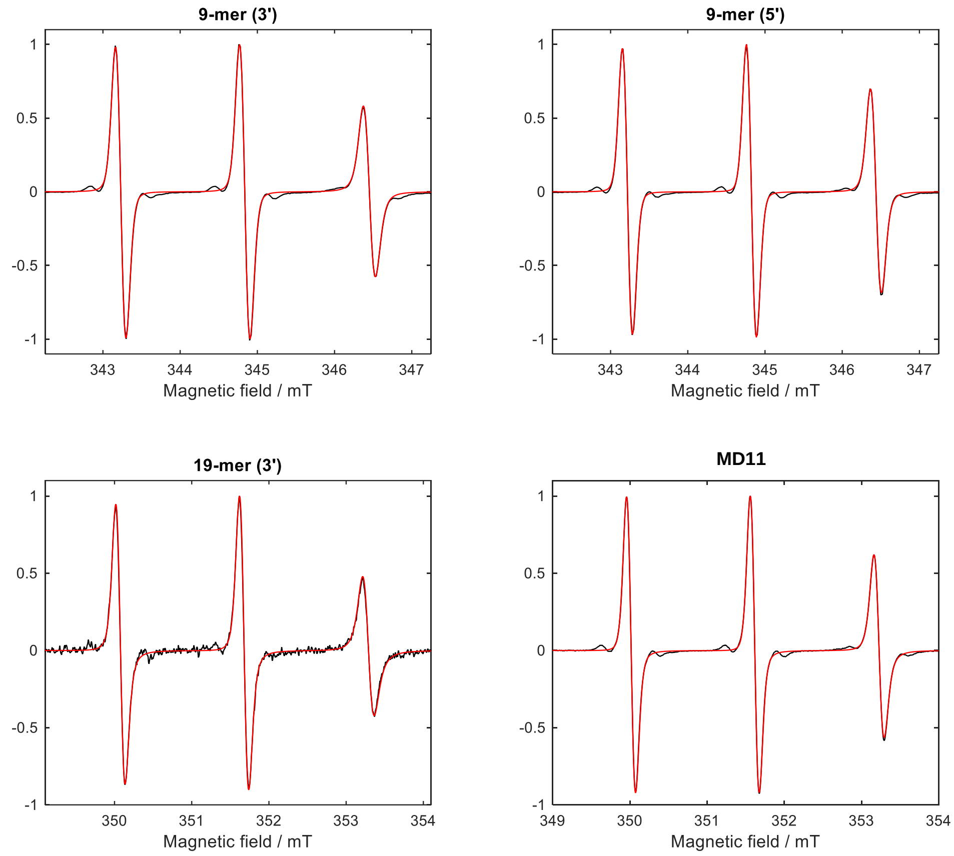

Figure S3. Room-temperature X-band CW EPR spectrum of MD11, 3-mer and 19-mer oligonucleotides in water. Black line – experimental, red line – simulated data.

DEER distance measurements

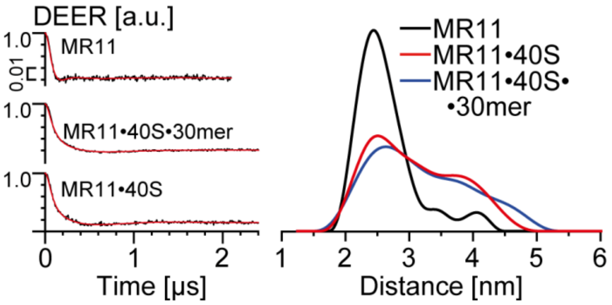

Figure S4. Distance measurements in the isolated MR11, in its binary mixture with 40S subunits and in its mixture with the ternary complex of 40S subunits with 30-mer mRNA UUCGACAACAAACACAACAAACGAAUAACA and P site $tRNA^{Asp}$, obtained by Q-band DEER.

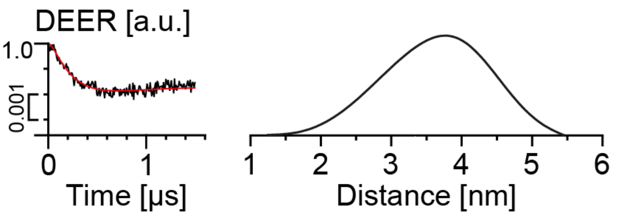

Figure S5. Distance measurements between 3’-label of 9-mer mRNA AAUAAAUAU and 3’-label of 19-mer mRNA GACUUCAACAAACACAACU being in complex with 40S subunits and P site $tRNA^{Asp}$, obtained by Q-band DEER.

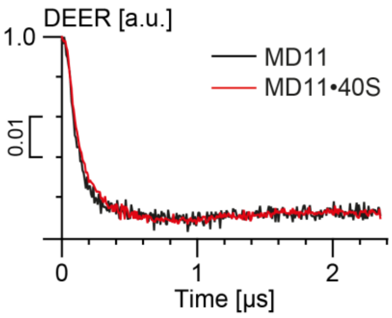

Figure S6. DEER time traces for MD11 and its binary mixture with 40S subunits after scaling to modulation depth.

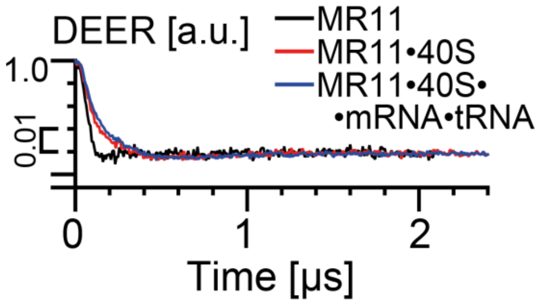

Figure S7. DEER time traces after scaling to modulation depth of isolated MR11, its binary mixture with 40S subunits and its mixture with the ternary complex of 40S subunits with 19-mer mRNA GACUUCAACAAACACAACU and P site $tRNA^{Asp}$.

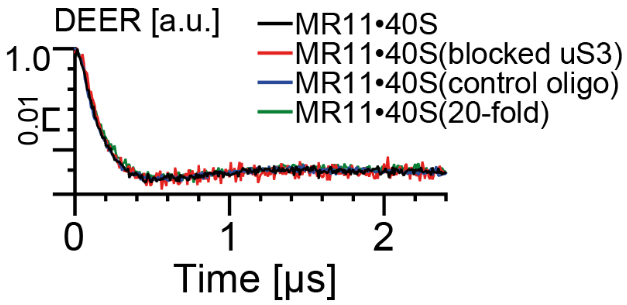

Figure S8. DEER time traces after scaling to modulation depth of MR11 with 40S subunits subjected to the same treatments as cross-linked without any additional components, with cross-linked 40S subunits and in the presence of unmodified nonaribonucleotide AAUAAAUAU or the 20-fold excess of the unmodified 11-mer UGUGUUCUAAA.

Raw traces and validation of DEER data

DEER time traces feature predominantly one full oscillation being enough for reliable conversion of a dipolar evolution into a distance distribution. However, since the mathematical problem is ill-posed, it is useful to validate distance distributions regarding to the influence of baseline correction procedure. Figure S9 represents data for all the samples, including raw time traces, Pake doublets (Fourier transforms of time traces) and corresponding distance distributions. All processing and error estimations were done using DeerAnalysis2016.

To validate the distributions, the starting time for background correction was varied within the 1000 ns around optimal time (11 trials), with resulting distributions indicating the errors. Error areas at the main part of distributions (2-5 nm) feature insignificant deviations, no peaks appearing or disappearing, which supports the fidelity of the solutions. Error areas also appear at 6 nm due to insufficient subtraction of a baseline and have nothing to do with genuine distances. Pake pattern

boxes in Figure S9 illustrate optimal cases of background subtraction, as well as under- and over-subtraction when zero-frequency component transforms to a spike or a deep hole, correspondingly.

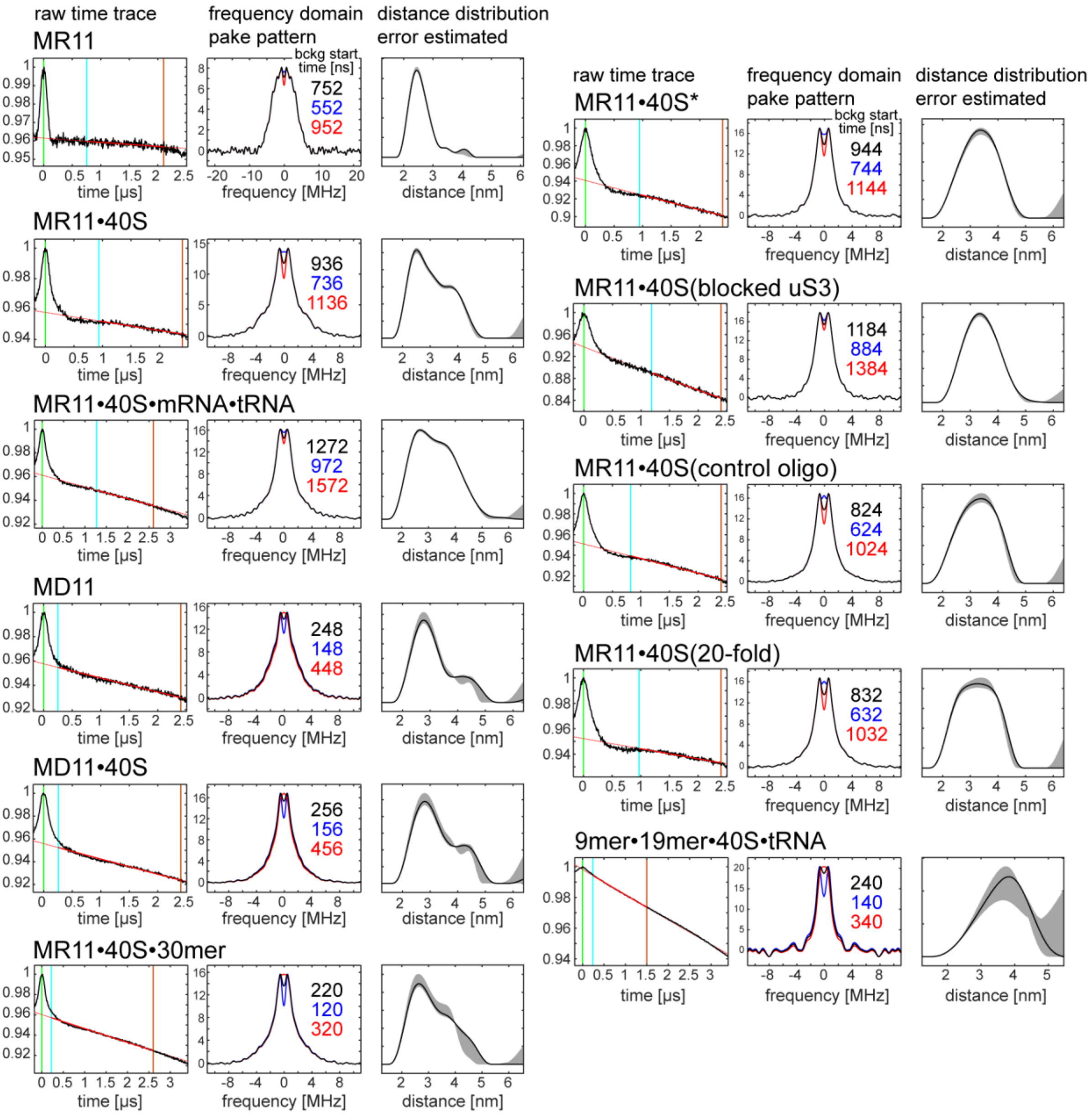

Figure S9. Raw DEER time traces, Pake patterns and distance distributions with error estimation for all considered samples. Raw traces are shown along with the vertical lines at positions of zero time (green), background starting time (blue) and cutoff (orange). Pake patterns are given for optimal starting times (black) and those deviating from the optimum of background correction by 100-300 ns. Distance distributions are supplemented with errors (grey area) estimated using DeerAnalysis2016 validation tool. MR11·40S* stands for the binary mixture of MR11 with 40S subjected to the same treatments as cross-linked without any additional components.
